# Longitudinal Multimodal Assessment of Structure and Function in *INPP5E*-Related Retinopathy

**DOI:** 10.3390/genes16121407

**Published:** 2025-11-26

**Authors:** Andrea Cusumano, Marco Lombardo, Benedetto Falsini, Michele D’Ambrosio, Jacopo Sebastiani, Enrica Marchionni, Maria Rosaria D’Apice, Barbara Rizzacasa, Francesco Martelli, Giuseppe Novelli

**Affiliations:** 1 Macula & Genoma Foundation, 00196 Rome, Italy; cusumano@cusumano.com (A.C.);; 2 Department of Experimental Medicine, Ophthalmology Unit, University of Rome Tor Vergata, 00133 Rome, Italy; 3 Macula & Genoma Foundation USA, New York, NY 10017, USA; 4 Medical Genetics Unit, Policlinico Tor Vergata, University of Rome Tor Vergata, 00133 Rome, Italynovelli@med.uniroma2.it (G.N.); 5 Department of Cardiovascular and Endocrine-Metabolic Diseases and Ageing, National Institute of Health, 00161 Rome, Italy

**Keywords:** inherited retinal dystrophy, multimodal imaging, retinal function, structure/function relationship, adaptive optics, *INPP5E* gene

## Abstract

**Background**: *INPP5E*-related retinopathy (*INPP5E*-RR) is a rare genetic disorder caused by biallelic pathogenic variants in the *INPP5E* gene, which encodes an enzyme critical for phosphoinositide signaling. While early-onset rod–cone dystrophy is a hallmark feature, detailed longitudinal data on the phenotype are scarce. This study aims to report a 6-year longitudinal assessment of retinal structure and function in a case of non-syndromic *INPP5E*-RR. **Methods**: A 42-year-old female proband with compound heterozygous pathogenic missense variants in *INPP5E* (p.Arg486Cys and p.Arg378Cys) was monitored from 2019 to 2025. She underwent serial comprehensive ophthalmologic evaluations, including optical coherence tomography (OCT), fundus autofluorescence, adaptive optics transscleral flood illumination, full-field 30Hz flicker electroretinography (ERG), and macular frequency-doubling technology perimetry. **Results**: Over the 6-year follow-up, OCT imaging revealed a progressive decline in the ellipsoid zone (EZ) width, from 1220 µm to 720 µm (~80 µm/year), and in the inner nuclear layer (INL) thickness. The central outer nuclear layer (ONL) thickness was preserved, but intraretinal cysts developed. Functional testing revealed a progressive decline in cone flicker ERG amplitudes, while visual acuity and macular perimetry remained stable. **Conclusions**: In this genotypically confirmed case, the longitudinal data identify EZ width, INL thickness, and cone flicker ERG as robust biomarkers of disease progression in *INPP5E*-RR. These parameters are ideal candidates for monitoring therapeutic outcomes in future clinical trials.

## 1. Introduction

*INPP5E*-related retinopathy refers to a rare genetic disorder caused by pathogenic variants in the *INPP5E* gene (MIM*613037). Visual symptoms often appear in childhood or adolescence. This gene encodes the inositol polyphosphate-5-phosphatase enzyme, which acts in the phosphoinositide signaling pathway within primary cilia [1].

The *INPP5E* pathogenic variants disrupt normal phosphoinositide signaling, leading to ciliary dysfunction characterized by abnormalities in the structure and function of photoreceptor cilia [1]. It is essential for the maintenance of the photoreceptor outer segment [2]. As a consequence of defective ciliogenesis resulting from disrupted signaling pathways, photoreceptor degeneration occurs, leading to the loss of photoreceptors [2].

Biallelic pathogenic variants (in homozygosity or compound heterozygosity) are associated with syndromic ciliopathies such as Joubert Syndrome type 1 (JBTS1 MIM #213300) and Impaired Intellectual Development, Truncal Obesity, Retinal Dystrophy, and Micropenis Syndrome (MORMS, MIM #610156). More recently, biallelic variants have been reported in non-syndromic inherited retinal diseases (IRDs) [3]. The prevalence of isolated INPP5E-related retinopathy is not precisely assessed. In a recently published cohort of Portuguese patients affected by retinal dystrophies of 130 index cases, the *INPP5E* gene was involved in 1.7% of solved and likely solved cases [4], whereas in a larger Italian cohort of 2970 patients affected by retinal dystrophies, *INPP5E* was implicated in 0.25% of the solved cohort [5].

While the phenotypes of non-syndromic *INPP5E*-related retinopathy have been described, including early-onset severe retinal dystrophy or milder juvenile-onset rod–cone degeneration [3], no information exists on their detailed retinal phenotype and natural history of retinopathy. The progression of disease is assumed to be relatively rapid, but no detailed evidence of this progression has been reported. Here, we report the longitudinal assessment over six years of a patient affected by non-syndromic *INPP5E*-related retinopathy.

## 2. Materials and Methods

### 2.1. Clinical Assessment

A 42-year-old female proband affected by non-syndromic IRD was monitored over a 6-year follow-up from 2019 to 2025 at the outpatient center of Macula & Genoma Foundation in Rome. She was first observed in 2019 at the age of 42, complaining of nyctalopia, reduced visual acuity, and restricted visual field. She underwent a comprehensive ophthalmological examination with multimodal imaging, including optical coherence tomography (OCT) and fundus autofluorescence (FAF) using the Spectralis system (Heidelberg Engineering, Heidelberg, Germany), with assessment of retinal layer thicknesses through the automatic segmentation of OCT B-scans. Ellipsoid zone (EZ) length was assessed by three different blind experienced operators with the Spectralis caliper function. Regarding visual function, best corrected visual acuity assessment with standard ETDRS charts, color vision assessed using the Ishihara pseudoisochromatic plates, International Society for Clinical Electrophysiology of Vision (ISCEV) standard full-field electroretinography (ERG) (Retimax, CSO, Florence, Italy), and frequency-doubling technology (FDT) perimetry (Humphrey Matrix, Carl Zeiss Meditec, Jena, Germany) were performed. Photopic 30 Hz flicker ERGs were also recorded using a submicrovolt technique [6]. Adaptive optics transscleral flood illumination (AO-TFI) was performed in a single session to capture high-resolution images at the cellular level of the photoreceptors (PRs) and retinal pigment epithelium (RPE) in the region of interest. Transscleral illumination of the retina [7] was performed using two near-infrared light-emitting diodes (wavelength, λ = 850 nm, pulse peak power = 250 mW per light-emitting diode, pulse duration = 8 ms, repetition rate = 11 Hz) located on the nasal and temporal side of the eye, coupled with an AO loop including a wavefront sensing (continuous illumination λ = 756 nm, peak power = 70 μW, maximum duration = 1800 s) embedded in a retina camera. Images were elaborated using custom-made software to produce high-resolution images at the cellular level of the PR and RPE for the region of interest. The patient was seen annually until 2021. Additional clinical follow-up was performed in our outpatient center in January 2025 and May 2025. The parents and a sister of the proband also underwent general and clinical examinations, including multimodal imaging and ERG.

### 2.2. Molecular Genetics

The proband was referred to the Medical Genetics Unit for genetic counseling after undergoing extensive, yet uninformative, genetic analyses. Beyond the retinopathy, she did not present any other systemic involvement at clinical examination. She was the second child of a non-consanguineous couple, with no remarkable family history of inherited diseases or a familial history of retinal diseases.

After written informed consent and in accordance with the Principles of the Declaration of Helsinki, genomic DNA was extracted from the proband’s and the parents’ peripheral blood mononuclear cells using the EZ1 Advanced XL Robotic workstation (QIAGEN, Hilden, Germany).

### 2.3. Whole-Genome Sequencing (WGS) and Data Analysis

Genomic DNA from the proband was prepared using the DNA PCR-Free Prep, Tagmentation^®^ Kit (Illumina, San Diego, CA, USA). Whole-genome sequencing was performed on the NovaSeq X Plus Sequencing System (Illumina, San Diego, CA, USA), achieving a mean sequencing depth of 30×. Bioinformatic processing was conducted using the DRAGEN Germline Pipeline (Illumina, San Diego, CA, USA) for read alignment and variant calling. Variant annotation and prioritization were carried out using GeneYX v6.2 (Geneyx Genomex Ltd., Herzlya, Israel) and NOSTOS Genomics software (AION 3.17.0.0) (Nostos Genomics GmbH, Berlin, Germany).

### 2.4. Sanger Sequencing

Primer pairs used to amplify fragments encompassing individual variants were designed using the online tool Primer3Plus (version 2.5.0) [8], and PCR amplifications were performed for each primer set according to the AmpliTaq Gold^®^ DNA Polymerase protocol (Applied Biosystems, Foster City, CA, USA).

Amplicons were bidirectionally sequenced using BigDye Terminator v3.1 Cycle Sequencing kit according to the manufacturer’s instructions on SeqStudio 8 Flex Genetic Analyzer (Applied Biosystems, Foster City, CA, USA).

## 3. Results

Clinical general examination was within normal limits. No systemic symptoms or signs were found.

### 3.1. Identification of Compound Heterozygous INPP5E Pathogenic Variants

**Figure 1:**
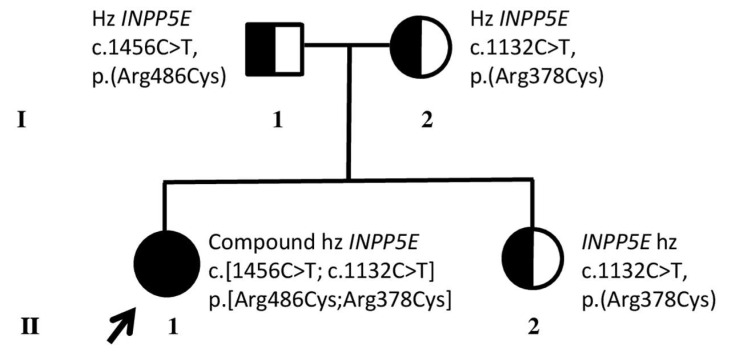
Family pedigree and molecular results. Black arrow and black circle indicate the proband (II.1) who underwent whole genome sequencing, resulting in compound heterozygous (hz) for *INPP5E* pathogenic variants. Half black circles and squares indicate her unaffected relatives (parents I:1, I:2) and sister (II:2), who resulted in hz for one of the *INPP5E* variants. I and II refer to the first and second generations of the family, respectively.

WGS analysis detected two heterozygous variants in the *INPP5E* gene: NM_019892.6:c.1132C>T, p.(Arg378Cys), classified as pathogenic (class 5), and NM_019892.6:c.1456C>T, p.(Arg486Cys), classified as likely pathogenic (class 4) following the American College of Medical Genetics and Genomics (ACMG) criteria [9]. Segregation analysis through Sanger Sequencing in the parents detected the first variant p.(Arg378Cys) in her mother (Figure 1, I:2) and the second variant p.(Arg486Cys) in her father (Figure 1, I:1), confirming the compound heterozygosity of *INPP5E*-related retinopathy variants in the proband. Segregation analysis in her unaffected sister (Figure 1, II:2) detected the p.(Arg378Cys) heterozygous variant.

The variant p.(Arg378Cys) is rare in the population database gnomAD (total allele frequency 0.00003384), and it is reported in the genomic database ClinVar (Variation ID: 400). It was previously reported in literature in JBTS1, and an in vitro enzymatic assay showed a slightly diminished activity in comparison to wild-type [10]. The variant has been more recently described in non-syndromic IRD [5]. The variant p.(Arg486Cys) is rare in the population database gnomAD (total allele frequency: 0.00008100) and is reported in the genomic database ClinVar (Variation ID: 391693). It has been previously described in JBTS1 [11] and as a recurrent variant in non-syndromic IRD [3,4]. Both variants localize to the inositol polyphosphate phosphatase catalytic domain, a cluster domain of many missense variants involving arginine residues, and are causative of IRD [3].

### 3.2. Longitudinal Structural and Functional Retinal Changes

**Figure 2:**
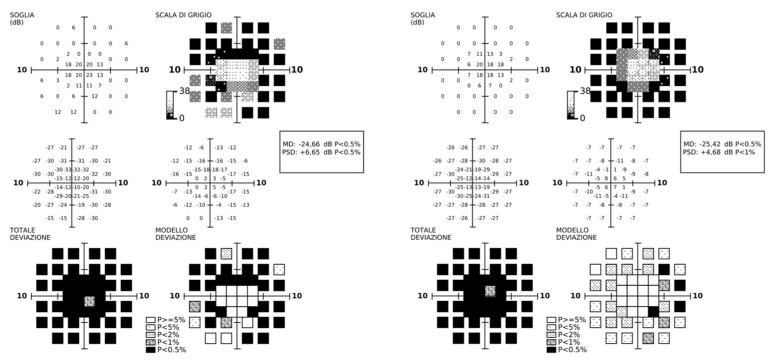
Central visual field. FDT perimetry showed a diffuse reduction in differential light sensitivity within the central 10 degrees compared to normative population values.

At diagnosis, visual acuity was 20/50 in both eyes, color vision was normal, and the visual field was restricted to a 15-degree diameter. The central visual field showed a diffuse reduction in perimetric sensitivity (Figure 2); the patient already had a compromised peripheral visual field.

Scotopic flash ERG amplitude was reduced to noise levels. Residual 30 Hz flicker responses recorded using a submicrovolt technique achieved a signal-to-noise ratio of 3 in both eyes.

**Figure 3:**
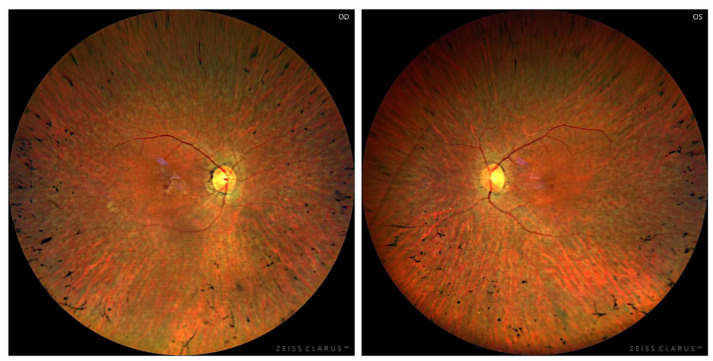
Fundus photography. Retinography showed features of typical retinitis pigmentosa (pale optic disc, thinning of retinal vessels, and pigmentation clumps in the mid-periphery).

Fundus examination showed features of typical retinitis pigmentosa with thinning of retinal vessels, pale optic disk, and pigmentation clumps in the mid-periphery (Figure 3).

**Figure 4:**
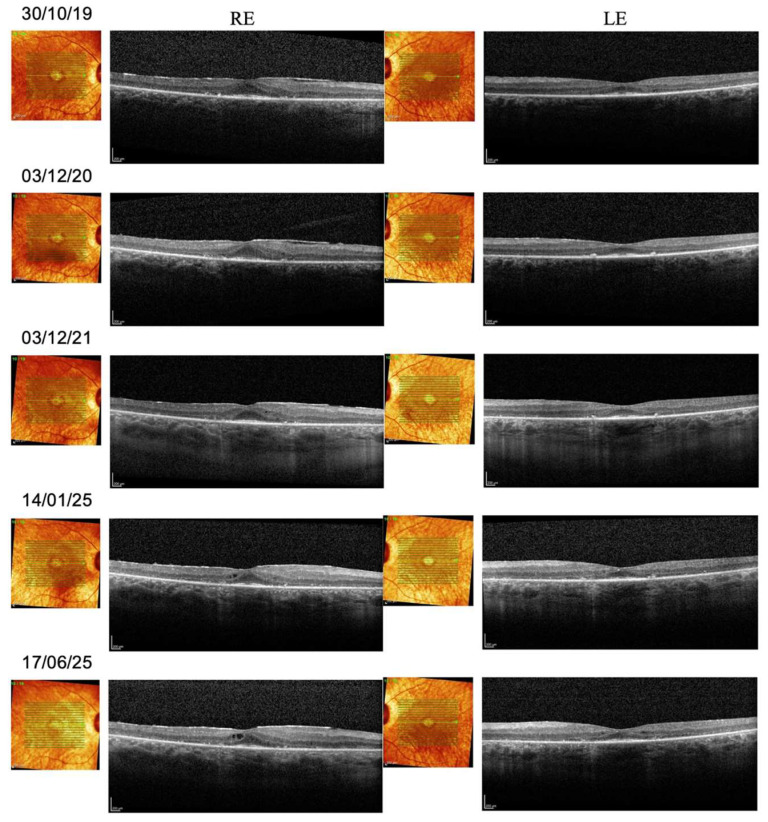
Spectral-domain OCT. Follow-up OCT imaging of the proband from 2019 (baseline) to 2025. RE: right eye; LE: left eye. A progressive thinning of the retina and a reduction in the length of the ellipsoid zone extension can be observed.

Spectral-domain OCT showed reduced thickness of the outer nuclear layer (ONL), which was nonetheless preserved in the foveal and parafoveal area. In the right eye, a microcystic degeneration was found. To date, no studies have reported the prevalence of intraretinal cysts specifically in *INPP5E*-related retinopathy. However, cystic macular changes are a recognized finding in other ciliopathy-associated IRDs [12,13]. There was a profound abnormality of retinal lamination, indicating retinal remodeling. The inner nuclear layer (INL) and ganglion cell layer (GCL) were below normal limits. Regarding longitudinal evaluation, central retinal thickness, which was reduced below normal values at baseline, did not show further decline over the follow-up period. In the right eye, central microcysts were stable. In both eyes, ONL thickness was stable. The EZ extension declined in both eyes, specifically from 1220 microns to 720 microns (a rate of 80 microns/year, assuming a linear progression) in the right eye, and from 931 microns to 748 microns in the left eye. The INL thickness declined progressively over the first three years of follow-up in both eyes and then stabilized. Altered inner retina lamination was progressive, with thinning of the GCL in both eyes. Figure 4 shows the OCT images taken from the proband during the follow-up period.

**Figure 5:**
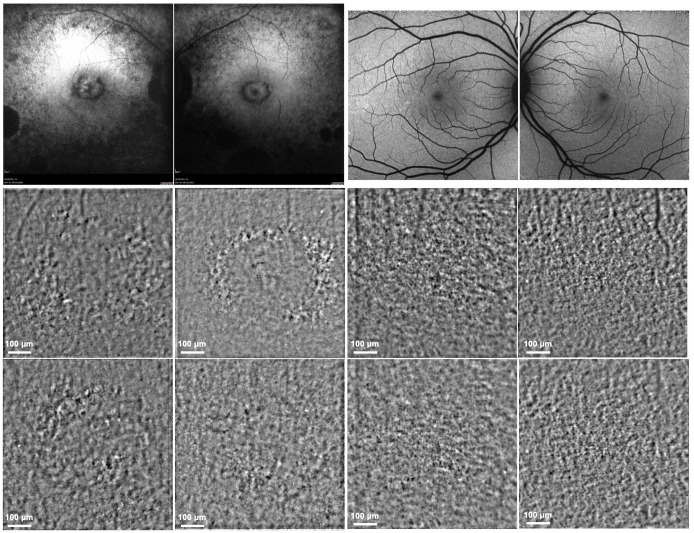
Fundus autofluorescence (FAF) and adaptive optics transscleral flood illumination (AO-TFI. FAF (**top**) and AO-TFI (**bottom**) imaging of the proband affected by *INPP5E*-related retinopathy (**left** panels) and of a healthy eye in comparison (**right** panels). FAF indicates diffuse atrophy of the outer retina with central circular atrophy of the retinal pigment epithelium (RPE); AO-TFI imaging displays alternating hypo- and hyperreflective regions that correspond to the pattern of RPE atrophy observed on FAF.

FAF showed extended areas of atrophy in the central retina and mid-periphery. The AO-TFI imaging revealed abnormalities in the RPE/PR with annular perifoveal patterns, which can be appreciated in comparison with the results of a normal control eye. In the same region of interest, FAF imaging obtained in the same examination session showed annular atrophy of RPE and outer retina in the region of interest (Figure 5).

**Figure 6:**
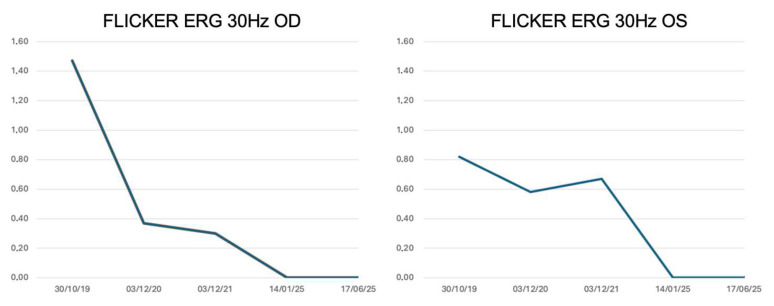
Electroretinography (ERG). Photopic flicker 30 Hz ERG (first harmonic) amplitude in microvolt plotted as a function of examination date. The graph shows a gradual yet consistent decrease over the years.

**Figure 7:**
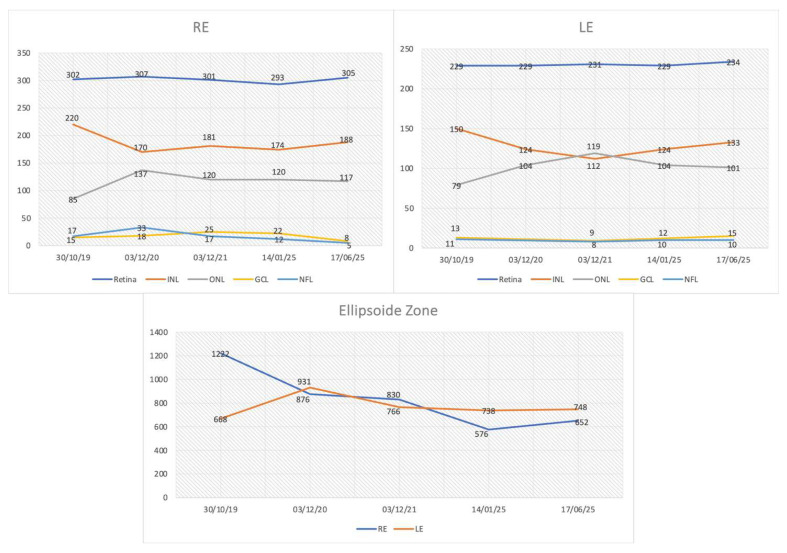
OCT parameters progression. Retinal layer thicknesses and ellipsoid zone (EZ) extension in microns plotted as a function of examination date. The parameter identified as experiencing the most significant decline over time was the EZ. RE: right eye; LE: left eye; INL: inner nuclear layer; ONL: outer nuclear layer; GCL: ganglion cell layer; NFL: nerve fiber layer.

Regarding visual function, visual acuity remained unchanged. The visual field showed a pericentral scotoma stable over time. The photopic flicker ERG showed small but detectable responses that declined progressively over time (Figure 6), correlating well with the morphological changes observed in the OCT (Figure 7).

Both parents and the proband’s sister had completely normal general and ophthalmic examinations.

## 4. Discussion

We present the first longitudinal characterization of retinal structure and function in a patient with non-syndromic *INPP5E*-related retinopathy, revealing key biomarkers of disease progression over a six-year follow-up. While biallelic pathogenic variants in *INPP5E* are typically associated with JBTS1or MORM syndrome, our case, compound heterozygous for two known pathogenic missense variants, underscores the existence of a milder, isolated retinal phenotype. This report expands the understanding of the natural history of this rare dystrophy, which was previously limited to cross-sectional descriptions.

Our multimodal approach uncovered a distinct pattern of progression. While visual acuity and macular perimetry remained stable, suggesting relative preservation of the central fovea, more sensitive metrics revealed ongoing degeneration.

All retinal layers derived from automated OCT segmentation and all standard full-field ERG parameters were initially examined. We then focused on three main biomarkers, EZ width, INL thickness, and 30 Hz cone flicker ERG amplitudes, because these were the only measures that showed a consistent and quantifiable change over the six-year follow-up, while other layer thicknesses and ERG components remained relatively stable or too close to noise levels to allow reliable longitudinal analysis. In addition, EZ width has been widely validated as a structural surrogate of photoreceptor integrity and a robust progression marker in retinitis pigmentosa and related IRDs [14,15,16]. INL thickness has been implicated as a marker of inner-retinal remodeling in degenerative retinopathies and in longitudinal structure–function studies of IRD [17,18]. Finally, 30 Hz cone flicker ERG provides a global, sensitive measure of cone pathway function and has been shown to correlate with disease progression and prognosis in retinitis pigmentosa and other inherited retinal disorders [19,20]. The combined use of EZ width, INL thickness, and cone flicker ERG, therefore, allowed us to capture outer retinal photoreceptor loss, inner retinal remodeling, and residual cone function within a coherent multimodal biomarker framework.

Specifically, we observed a rapid, linear decline in the EZ width at a rate of approximately 80 µm/year. This rate is consistent with EZ constriction reported in other rod–cone dystrophies, including RPGR-associated retinitis pigmentosa, where annual losses of 4–10% have been described and are considered robust structural endpoints in natural history and clinical trial studies [15,21,22]. This structural loss in the photoreceptor layer was paralleled by a progressive functional decline, as evidenced by the reduction in cone flicker ERG amplitudes. This combined structural–functional pattern aligns with recent evidence showing that cone flicker ERG is among the most sensitive functional parameters to detect progression in rod–cone dystrophies [23]. Furthermore, we documented a progressive thinning of INL and GCL, indicating a substantial remodeling of the inner retina that extends beyond the primary photoreceptor pathology. The novel application of AO-TFI imaging provided a high-resolution view of the abnormalities in the RPE/PR complex in the perifoveal region, anatomically explaining the sensitivity loss detected by perimetry.

The dissociation between stable visual acuity and progressive decline in EZ width, INL thickness, and cone ERG is clinically significant. It demonstrates that these quantitative measures are more sensitive indicators of disease progression than standard functional tests alone. This structure-function relationship has not been previously detailed in *INPP5E*-related retinopathy.

The stability of visual acuity and macular perimetry contrasts with the progressive decline detected by more sensitive structural and electrophysiological metrics. This apparent preservation can be explained by the relative sparing of the foveal region, as shown on OCT: the foveal ONL and EZ remained intact throughout follow-up. In rod–cone dystrophies, foveal cones usually survive longer than parafoveal photoreceptors, resulting in preserved central visual acuity despite significant extra-foveal degeneration.

Similarly, macular perimetry was already markedly depressed at baseline, and psychophysical tests such as FDT perimetry have limited dynamic range and reduced sensitivity for detecting small changes once retinal sensitivity approaches floor values. In contrast, quantitative OCT-derived metrics and 30 Hz cone flicker ERG amplitudes can detect subtle structural or functional changes. This may explain the observed dissociation between stable central vision and progressive abnormalities captured by these more sensitive biomarkers.

Currently, no treatments exist for *INPP5E*-related retinopathy. However, the development of future therapies, such as gene therapy, will depend on a precise understanding of the natural history and the identification of robust biomarkers for clinical trials. Our findings posit that the EZ width, INL thickness, and cone flicker ERG are excellent candidate biomarkers for monitoring disease progression. They offer a sensitive toolset to identify the optimal therapeutic window, particularly in juvenile forms of the disease, and to objectively evaluate treatment efficacy in future interventional studies.

This study has limitations. It is based on a single-case longitudinal observation, which restricts generalizability to the broader *INPP5E*-related retinopathy spectrum. Additionally, mesopic vision testing was not performed because these retrospective clinical data did not include this assessment.

## 5. Conclusions

This longitudinal study provides the first in-depth natural history of non-syndromic *INPP5E*-related retinopathy, combining multimodal imaging and electrophysiological assessments over a six-year period. The findings demonstrate a progressive yet measurable structural and functional decline characterized by EZ shortening, inner retinal thinning, and reduction in cone flicker ERG amplitudes, despite relative stability of central visual acuity. These results identify quantitative biomarkers, particulsarly EZ width, INL thickness, and cone ERG amplitude, as sensitive indicators of disease progression. Overall, this case contributes valuable insight into the pathophysiology of *INPP5E*-related retinopathy and provides a reference framework for monitoring treatment efficacy in forthcoming gene-based therapeutic studies.

## Data Availability

The data presented in this study are available upon request due to restrictions (privacy reasons) from the corresponding author.

## Abbreviations

The following abbreviations are used in this manuscript:

JBTS1	Joubert Syndrome type 1
MORMS	Impaired Intellectual Development, Truncal Obesity, Retinal Dystrophy, and Micropenis Syndrome
IRD	Inherited retinal disease
OCT	Optical coherence tomography
FAF	Fundus autofluorescence
EZ	Ellipsoid zone
ERG	Electroretinography
FDT	Frequency-doubling technology
AO-TFI	Adaptive optics transscleral flood illumination
PR	Photoreceptor
RPE	Retinal pigment epithelium
WGS	Whole-genome sequencing
ONL	Outer nuclear layer
INL	Inner nuclear layer
GCL	Ganglion cell layer
RE	Right eye
LE	Left eye
